# Reconciling Estimates of Ocean Heating and Earth’s Radiation Budget

**DOI:** 10.1007/s40641-016-0053-7

**Published:** 2017-01-16

**Authors:** Matthew D. Palmer

**Affiliations:** Met Office Hadley Centre, FitzRoy Road, Exeter, UK

**Keywords:** Ocean heat content, Earth’s energy imbalance, Earth’s radiation budget, Argo, Ocean observations, Satellite observations, CERES, Climate change, Ocean heating

## Abstract

**Purpose of review:**

The purpose of this review is to summarise the recent literature and scientific challenges on the topic of reconciling estimates of ocean heating rates with satellite-based monitoring of Earth’s radiation budget (ERB), including discussion of the satellite record and in situ ocean observing system.

**Recent findings:**

State-of-the-art climate model simulations suggest that the global ocean becomes the dominant term the planetary heat budget on annual and longer timescales. Therefore, we expect to see a close correspondence between year-to-year variations in ocean heating rates and satellite measurements of ERB. Recent comparisons of satellite ERB time series and ocean heating rates show a marked improvement over earlier studies in terms of consistency and specification of uncertainties. Contemporary research has also emphasised the utility of these independent data sets for cross validation of the climate record and their fundamental importance for monitoring the rate of climate change.

**Summary:**

Anthropogenic greenhouse gas emissions have brought about an imbalance in Earth’s radiation budget that is driving global climate change. Our primary means for monitoring this energy imbalance is via direct satellite measurements of ERB and through estimates of global ocean heat content (OHC) change. CERES satellite measurements of ERB offer high spatiotemporal resolution and uncertainties on annual time series of order 0.1 Wm^-2^ but cannot provide absolute monitoring of Earth’s energy imbalance due to limitations in sensor calibration. The Argo array of autonomous profiling floats has revolutionised the ocean observing system and our ability to estimate absolute ocean heating rates with current uncertainties estimated to be 0.5/0.1 Wm^-2^ on annual/decadal timescales. These ocean observations are essential to “anchor” the time series of ERB and can be used to mitigate satellite sensor drifts. Sustaining these highly complementary elements of the climate observing system is essential for improved understanding of climate variability and change. Improvements in satellite sensor calibration, estimates of total solar irradiance and more comprehensive sampling of the global oceans (e.g. Deep Argo) are key aspects to reducing uncertainties in future observations of Earth’s energy imbalance.

## Introduction

All of the energy that enters or leaves the Earth system does so radiatively at the top of Earth’s atmosphere. For an equilibrium climate, the absorbed solar radiation (the total incoming short wave radiation minus that which is reflected back into space by Earth’s albedo) is balanced by the planetary emitted long wave (LW) radiation, over some suitable long-term average. Anthropogenic global warming arises from elevated greenhouse gas concentrations that lead to a persistent imbalance in Earth’s radiation budget (ERB) and an accumulation of thermal energy in the Earth system, which is the root cause of the various facets of observed climate change [1]. This picture is complicated by substantial short-term variations in the net radiation at top-of-atmosphere, owing to internal weather and climate variability within the Earth system [2–5].

On multi-decadal timescales, it is estimated that >90% of the planetary heating associated with Earth’s energy imbalance (EEI) goes directly into warming of the global oceans, with much smaller amounts going into heating of the land, atmosphere and ice cover [1, 6]. Climate models suggest that the global ocean becomes the dominant term in Earth’s energy budget on timescales longer than about 1 year [4]. Therefore, we expect to see a very close correspondence between the rate of ocean heat content (OHC) change and variations in ERB on interannual and longer timescales.

Two particular developments have played a substantial role in promoting research into EEI over the last few years. Firstly, advances in the global climate observing system over the last 15 years have brought about a step change in both our ability to monitor variations in ERB using satellites [7] and our ability to estimate OHC changes using the Argo array of autonomous profiling floats [8]. Secondly, the widespread discussion around the global surface warming slowdown, or “hiatus”, has motivated researchers to better understand the mechanisms of global surface temperature (GST) variability and linkages to EEI (e.g. [9–11]). One of the important recent discoveries, which has been elucidated by both observational [12] and climate model [4, 13] studies, is the decoupling of GST trends and EEI on decadal timescales. Thus, the idea that the recent slowdown in surface temperature rise signalled the end of anthropogenic climate change is based upon a false premise.

There are two primary means by which we can make observational estimates of EEI (see [1] for a review of various approaches). The first is to make use of direct satellite measurements of variations in ERB at the top-of-atmosphere. The second is to estimate changes in the global energy inventory, which on interannual and longer timescales is dominated by changes in OHC [4, 6, 13]. These two approaches are highly complementary, making use of totally independent data sets, and their cross validation provides an important means to detect systematic errors in each observational system. Substantial advances in satellite and ocean in situ observing capabilities have come about since the early 2000s via the NASA Clouds and the Earth’s Radiant Energy System (CERES) project and the Argo global array of autonomous profiling floats [8], respectively. Attempts to reconcile estimates of EEI from TOA radiation measurements and OHC change have therefore mostly focussed on these two data sources.

In the next section, we present a brief review of satellite ERB measurements, discuss the capabilities of CERES observations and an effort to reconstruct a continuous record of ERB back to 1985. This is followed by sections that discuss the main challenges in estimating OHC change from in situ temperature observations and research efforts that attempt to reconcile ERB measurements with ocean heating rates. In the final section, I present my conclusions and some thoughts on potential future research avenues.

## Estimates of Earth’s Radiation Budget

**Fig. 1:**
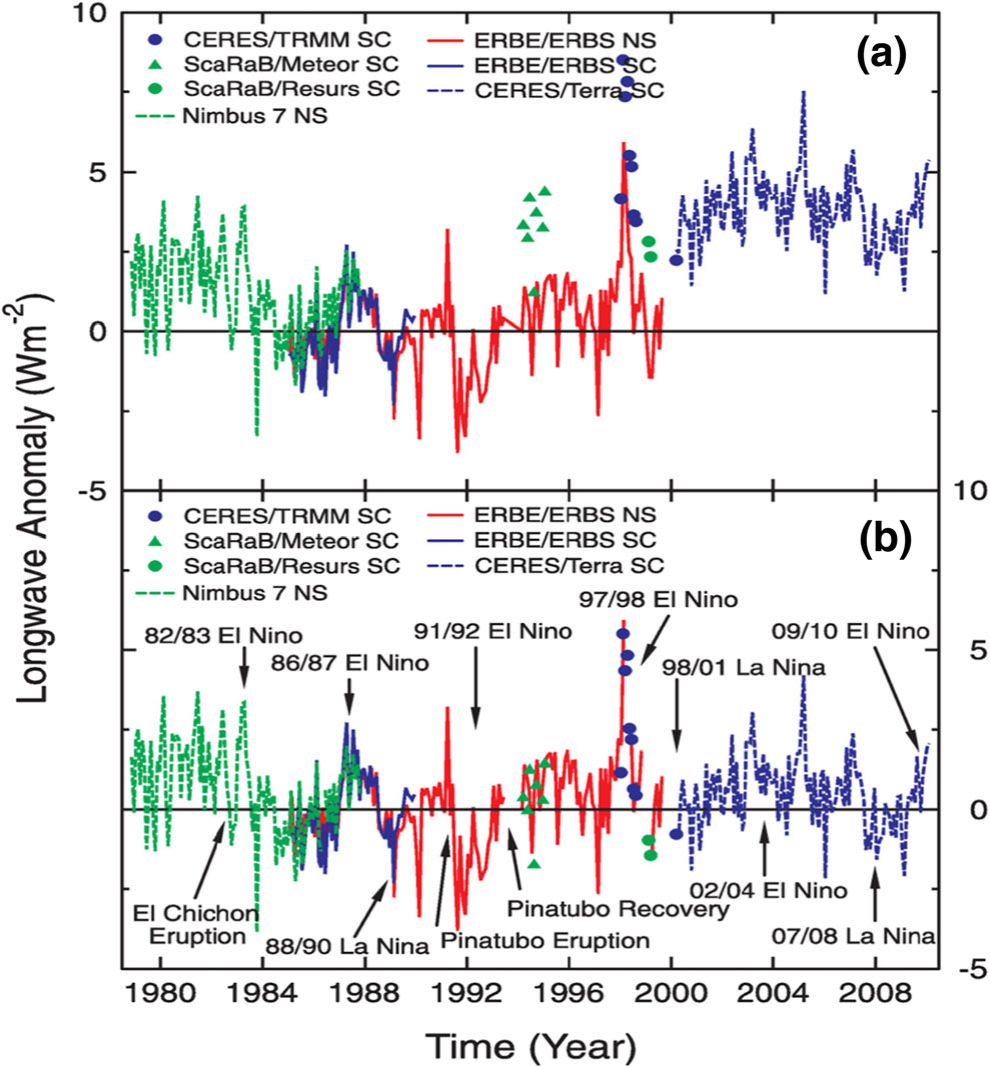
Long wave (LW) top-of-atmosphere flux anomalies for 20°S–20°N from November 1978 to February 2010 **a** with no overlap correction and **b** with overlap correction based upon ERBS Nonscanner WFOV Edition3_Rev1 (*red solid line*), Nimbus-7 Nonscanner (*green dashed line*), ERBS Scanner (*blue solid line*), CERES Terra crosstrack SSF1deg-lite_Ed2.5 (*blue dashed line*), CERES/TRMM Scanner Edition2 (*blue circle*), ScaRaB/Meteor Scanner (*green triangle*) and ScaRaB/Resurs Scanner (*green circle*). Anomalies are defined with respect to the 1985–1989 period. Reprinted with permission from Loeb et al. [2]

While the first satellite observations of ERB at the top-of-atmosphere came during the 1960s (e.g. [14]), systematic monitoring of the radiative components only began in the late 1970s with Nimbus-7 [15]. As an illustration of successive satellite missions to monitor ERB, we show the continuous 31-year record of tropical outgoing LW radiation between 1979 and 2010 published by Loeb et al. [2] (Fig. 1). The record shows marked jumps between successive satellite products, owing to differences in the absolute calibration of the instruments (Fig. 1a). However, these differences can be accounted for during overlap periods and the entire record placed on a common scale (Fig. 1b). The complete record shows large variations in tropical outgoing long wave radiation, with peak values of ±5 Wm^−2^ that are usually associated with major volcanic eruptions or large ENSO activity and substantial decadal variability [16].

The current state-of-the-art satellite measurements come from NASA’s CERES project, with the primary measurements of outgoing total and short wave radiances measured by scanning instruments on the *Terra* and *Aqua* satellites. Daytime long wave radiance is determined through subtracting short wave radiance from total radiance. Nighttime long wave radiance is based solely on total radiance. These long wave and short wave radiances are then converted into radiative fluxes using angular dependence models. CERES makes use of the SORCE measurements of total solar irradiance [17] and several other data satellite sources in the data processing, as described by Loeb et al. [7]. CERES has now provided over 16 years of continuous ERB measurements with unprecedented sensor accuracy, stability and well-resolved spatial information (1 × 1 degree) for each radiative component [2, 7]. Particular advances over predecessor products based on NASA’s Earth’s Radiation Budget Experiment (ERBE) include use of consistent cloud and aerosol properties from moderate resolution imaging spectrometer (MODIS) and new empirical angular distribution models [18]. However, limitations in absolute sensor calibration accuracy mean that CERES measurements must be adjusted in order to close the energy budget in absolute terms (e.g. [7, 19]).

Loeb et al. [7] have carried out a detailed assessment of the uncertainties (reported at 5–95% confidence level) based on 1 × 1 degree monthly averages for the period March 2000 to February 2005. The authors report that the largest sources of uncertainty are from instrument calibration (4.2 Wm^−2^) and the assumed value for total solar irradiance (1 Wm^−2^). The uncertainty associated with errors in time and space interpolation, which are assessed by comparing differences between measurements from the *Terra* and *Aqua* satellites, is estimated as ±0.3 Wm^−2^. The sensor stability has been assessed as better than 0.5 Wm^−2^ per decade, also based on comparisons between *Terra* and *Aqua* and a number of additional satellite data products. Therefore, despite the inability to resolve the absolute value of EEI, CERES observations provide invaluable information on the spatiotemporal variations in EEI and its radiative components. These observations are particularly powerful when combined with vertical cloud and aerosol profile data from CloudSat [20] and CALIPSO [21], in order to understand the processes that give rise to variations in ERB.

In general, satellite estimates of geophysical variables rely on a series of models and assumptions and are subject to reprocessing when sensor drifts, orbital changes or other problems are discovered (e.g. [2, 22]). Inter-satellite calibration is also a particular issue for a homogeneous climate record, and efforts must be made to ensure enough overlap is achieved between successive missions to properly account for differences in sensor characteristics. The CERES estimated sensor stability of 0.5 Wm^−2^ is not really adequate for climate change studies, since the drift over a decade is a similar magnitude to the climate change signal of 0.5–1 Wm^−2^ [5, 23] and therefore implies a role for “anchoring” ERB measurements with ocean heating rates.

Allan et al. [24] have used a number of model and satellite data sources, including the ERA-Interim atmospheric reanalysis, with the aim of creating a consistent and continuous record of ERB from 1985 to 2012, based upon ERBE and CERES data sets. In addition, simulations from high resolution (25 km) atmospheric simulations were used to bridge two gaps in the ERBE record, during 1999–2000 and 1993. Ultimately, any satellite-based reconstruction must be “anchored” with estimates of ocean heating rates, so it is the *variations* in EEI rather than its absolute value that are of primary relevance. The results show that the Mount Pinatubo eruption presents the largest perturbation to EEI over the study period (−3 Wm^−2^ peak, based on monthly mean data) and substantial decadal variability at other times (about ±0.5 Wm^−2^). While EEI was elevated over the period 1994–1998, following the Pinatubo eruption, there is little evidence of a reduction in EEI during the early 2000s “hiatus” period. Overall, the study highlights the large variations in EEI that are associated with volcanic forcing and tropical ENSO variability that present a challenge for monitoring the anthropogenic influence on EEI on decadal and shorter timescales.

## Estimates of Ocean Heating Rates

Abraham et al. [25] have reviewed the evolution of the historical ocean temperature measurement systems, sampling characteristics and implications for estimating changes in OHC. More recently, Desbruyères et al. [26] have provided a review focussed on twenty-first century ocean measurements for insights into the planetary energy and sea level budgets. We refer the reader to these papers for a more extended discussion of this topic and limit our attention to summarising the key challenges for OHC change estimates and some of the most promising approaches.

Prior to the inception of the Argo array of profiling floats in the early 2000s, the majority of ocean temperature profiles (the “building blocks” of estimates of ocean heating rates) came from expendable bathythermograph instruments (XBTs) and are limited to the upper few hundred metres. As a result, estimates of OHC change that extend back to the mid-twentieth century tend to be limited to the 0–700 m layer, which represents only the upper 20% of the average open ocean depth. Intercomparisons of upper ocean OHC change time series from both statistical approaches [5, 25, 27–29] and ocean data assimilation models [30–32] have shown large variations among the estimates in terms of both multi-decadal trends and interannual variations. As discussed by Palmer et al. [29], differences among OHC change estimates essentially arise from three sources: (1) input data and quality control, (2) correction of inter-platform data biases and (3) the mapping method used to infill data. Elements (2) and (3) have emerged as the leading uncertainty terms in estimates of OHC change [27, 28] and are the focus of the discussion for the rest of this section. Exploring the impact of (1) on OHC estimates remains an outstanding research challenge, and insights may be offered by the ongoing intercomparison of automated quality control checks under the International Quality Controlled Database initiative (IQuOD; [33]).

The impact of inter-platform biases on historical ocean heating rates was highlighted by Gouretski and Koltermann [34]. While the authors also documented systematic biases in the earlier mechanical bathythermograph (MBT) data, it is the effect of XBT biases that are more important for estimates of ocean heating rates, due to the large number of profiles and longevity of this instrument in ocean monitoring. XBTs account more than 50% of ocean profile data between 1967 and 2001. The time-space varying temperature biases associated with XBTs arise from a number of sources, including the fall-rate equation that is used to estimate the probe depth as a function of time [25]. While the first attempts to correct XBT biases were able to eliminate major differences among observed and simulated global ocean heat uptake and sea level rise [35], new correction schemes continue to be developed [36, 37] under ongoing research by the international community.

A major challenge in efforts to refine bias correction schemes is the lack of metadata for XBT instruments. For example, approximately 50% of XBTs in the historical databases are of unknown type. This has required researchers to make intelligent guesses about the likely probe type, based on information from individual profiles, such as the maximum recorded depth, country of origin and profile date [36, 37]. Efforts to devise more comprehensive approaches to missing metadata and assess its impact on ocean heating rates are being fostered through international collaborative projects, such as the IQuOD (www.iquod.org; [33]).

**Fig. 2:**
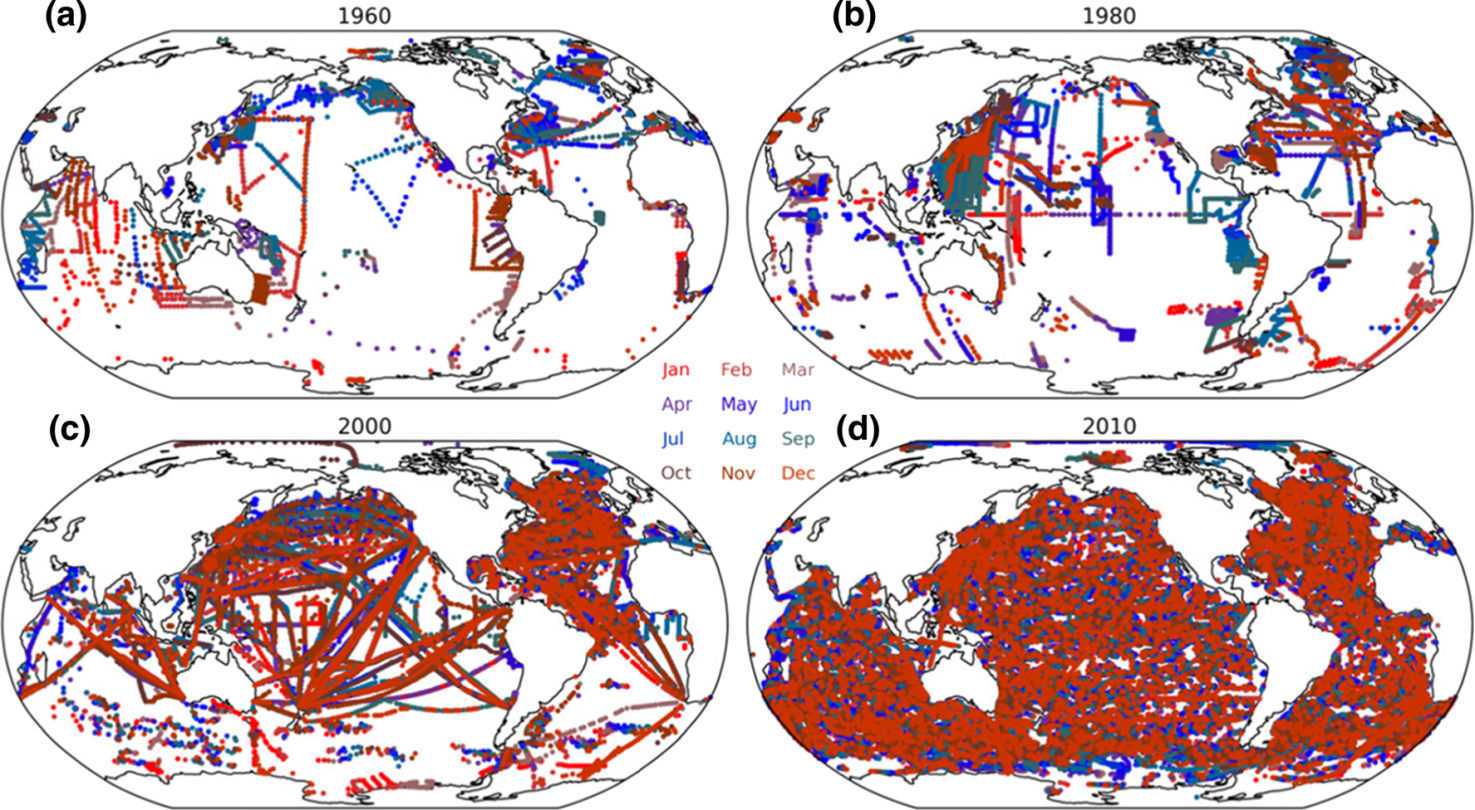
Sampling of the 0–700-m ocean based on EN3 temperature profiles [45] for four example years. *Colours* indicate the month in which the profile was recorded. Figure courtesy of Simon Good

A wide variety of approaches have been used in mapping temperature profiles over the full ocean domain in order to estimate both global and regional OHC changes. These broadly fall into two categories: (1) those that take a statistical approach to temporal and spatial infilling and (2) those that are based on an ocean reanalysis (ORA), which make use of a dynamical ocean model and some form of data assimilation scheme. Statistical approaches range from simple grid-box averaging of the data (e.g. [34, 38]) to various objective mapping or optimal interpolation approaches (e.g. [39, 40]) and schemes that estimate, and make use of, global relationships among grid boxes (e.g. [35, 41]). Climate change studies and climate monitoring activities have tended to focus on statistical estimates as the primary means for assessing observed OHC change (e.g. [6, 42]). ORAs vary in the variety of data inputs, the type of data assimilation scheme, the underlying model physics and imposed model boundary conditions (e.g. [31]). Data assimilation approaches are attractive because they are able to provide a dynamically consistent estimate of the ocean state. However, ORAs are subject to the limitations of the underlying model physics and data assimilation methods, and further work is needed to understand inter-product differences and improve their utility for estimating OHC change and other climate applications [31, 43]. Whichever mapping method is used, a fundamental limitation on estimates of historical ocean heating rates is the number, and sampling characteristics, of historical ocean temperature profiles (Fig. 2).

While the earliest subsurface ocean observations date back to eighteenth and nineteenth centuries, it was not until the advent of the XBT instrument in the late 1960s that routine widespread sampling of the upper ocean became possible. Ocean temperature observations over the latter half of the twentieth century are generally sparse and tend be clustered along shipping routes, with many fewer observations in the Southern Hemisphere and extremely limited sampling of the Southern Ocean (Fig. 2; e.g. [25]). Deployment of XBTs from the late 1960s dramatically improved both the total number and geographic coverage of ocean profiles and marked the first time that sampling was adequate for some assessment of global ocean heat content change in the upper 700 m or so [44]. Coverage and depth sampling of the ocean improved over time as XBT instruments were developed that could sample to 1 km and deeper (Fig. 2b, c; [37]). The early 2000s saw a dramatic improvement in ocean sampling with the development of the Argo array of autonomous profiling floats (Fig. 2d; [8]). The array reached its target population of 3000 active floats in November 2005, with each instrument profiling the upper 2 km of the water column and transmitting the data back in real time on a ten-day repeat cycle.

Argo represents a new era of quasi-global ocean sampling and the high-quality CTD systems used on the floats (and careful delayed-mode quality control) means that the problem of inter-platform biases is largely eradicated. As a result, the Argo period (from about 2005 or so) has become the focus of comparisons of ocean heating rates with satellite measurements of variations in ERB (see following section). However, even during this “golden age” of ocean sampling, there are substantial differences among estimates of ocean heating rates that arise from different mapping approaches used to provide spatially complete fields from single-point profile observations in a turbulent ocean [8, 12]. Although Argo samples many marginal seas and is expanding to better observe seasonally ice-covered areas (such as the Weddell and Ross Seas), these remain relatively poorly observed regions [1]. The core floats of the global array do not sample the ocean below 2 km (approximately the upper 50% of the open ocean depth). However, a few Deep Argo floats are currently being deployed by several countries in small regional pilot arrays (www.argo.ucsd.edu). Further research efforts are needed to assess the relative importance of these under-sampled areas and the impact for estimates of total ocean heating rates.

## Attempts to Reconcile Estimates of ERB and OHC Change

The first attempt to confront satellite measurements of variations in ERB with ocean heating rates was carried out by Wong et al. [46]. The authors compared two relatively short and separate time series from (1) ERBE/ERBS Nonscanner WFOV and (2) CERES, with the OHC change estimates of Willis et al. [47]. While the analysis was largely qualitative, e.g. there was no attempt to quantify the uncertainties for the different time series, the comparison was favourable. For example, both the satellite measurements and OHC estimate suggested a similar increase in planetary heating of 1.0–1.5 Wm^−2^ over the period 1994–1998 before a large drop of the same magnitude over 1998–1999, associated with the large 1997–98 El Nino event [47].

Trenberth and Fasullo [48] highlighted the importance of tracking Earth’s energy imbalance in a controversial paper that compared the estimates of ERB from CERES [22] with an estimate of OHC change [40] and the energy flux associated with melting of land-based ice (shown to be a very small term). The paper reported a large discrepancy between ERB and OHC change estimates, with the “missing energy” indicating a failure to close the Earth energy budget. However, Wong et al. [22] noted that the CERES data from late 2007 to 2009 was based on the preliminary “FLASHFlux” dataset with possible instrument stability artefacts. The Levitus et al. [40] OHC change estimate used by Trenberth and Fasullo [48] was also a subject to large uncertainties, as illustrated by comparative studies of ocean heating rates (e.g. [25, 28, 29]), which likely also played a part in the reported discrepancy. The paper also provided a useful comparison with auxiliary data sets, including global mean sea level, which is closely linked to OHC change via thermal expansion [49] and motivated several subsequent studies.

Loeb et al. [3] carried out their own analysis of observation changes in ERB and ocean heating rates for the period 2001–2010, making use of three different OHC estimates [38, 40, 44] and a reprocessed CERES satellite data set. A key advance in this paper was the provision of error estimates on all reported time series. A comparison of ocean heating rates for the period 1993 to 2010 showed large variations among the data products, but consistency within the (large) estimated uncertainties for both interannual and longer-term changes (1993–2003 and 2004–2008). The PMEL/JPL/JIMAR OHC product [44] was used as the basis of comparison with CERES ERB variations, and the two times series again showed consistency within the estimated uncertainties. The work highlighted the large sampling uncertainties for interannual ocean heating rates (1–2 Wm^−2^), which were an order of magnitude larger than the satellite measurements (0.1–0.3 Wm^−2^). The paper also illustrated that the radiative variations observed by CERES were well simulated by the ERA-Interim atmospheric reanalysis [50]. Loeb et al. reported an EEI of 0.50 ± 0.43 Wm^−2^ based on the upper 1800 m OHC change for the period 2001–2010.

Trenberth et al. [5] have highlighted the potential for ocean reanalyses to provide useful estimates of OHC change, using the ECMWF ORAS4 product [51]. In particular, the authors cite ORAS4’s clear response to negative radiative forcing from volcanic eruptions and its ability to provide an estimate of full-depth OHC change as advantages over statistical approaches. The ORAS4 estimate for EEI is relatively large at 0.91 ± 0.1 Wm^−2^ averaged for the period 2000–2010. However, substantial discrepancies between OHC change estimates and CERES measurements were shown at interannual timescales, with the period 2008–2009 being particularly problematic. One of the issues discussed in the study is the method used to estimate the rate of ocean heat content change and the need to provide some smoothing to reduce the noise.

**Fig. 3:**
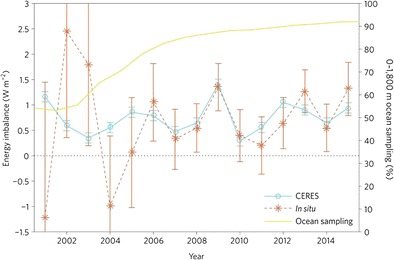
Comparison of year-to-year net top-of-the-atmosphere annual energy flux from the CERES Energy Balanced and Filled (EBAF) Ed2.8 product with an in situ observational estimate of uptake of energy by Earth’s climate system. The in situ estimate (*orange)* is based on the first difference of Argo annual ocean heat content estimates and a constant heating rate assumed for the deep ocean and other energy stores. The uncertainty bars indicate one standard error of the mean. CERES data (*blue*) have been adjusted to match the in situ heating rate of 0.71±0.1 Wm^−2^ for the period 2005–2015. CERES annual random errors are shown at one standard deviation (0.1 Wm^−2^). The percentage volume of ocean for 0–1800 m layer is indicated by the *yellow line*. Reprinted with permission from Johnson et al. [23]

Following Loeb et al. [3], Johnson et al. [23] have provided a more comprehensive assessment of planetary heat storage changes, including estimates for the ocean heating below 2000 m, melting ice and warming of the land and atmosphere. They have updated the comparison of CERES and ocean heating rates for the period 2001–2015, showing a greater correlation and a dramatic reduction in uncertainty for OHC that is primarily due improved Argo sampling through time (Fig. 3). The study appears to show a substantially improved agreement between CERES and OHC changes compared to Trenberth et al. [5], particularly from 2006 onwards. However, since both the CERES and OHC change estimates were updated between these studies, it is hard to pin down the origin of the improvement. One possible factor is that delayed mode quality control of Argo observations can take a few years to carry out, so the most recent estimates of OHC change may improve over time. Johnson et al. [23] estimate a time-average EEI of 0.71 ± 0.09 Wm^−2^ with uncertainties in interannual heat content change of about 0.5 Wm^−2^ following the completion of the Argo array in late 2007. The corresponding uncertainty in annual mean ERB from CERES is estimated to be about 0.1 Wm^−2^.

Smith et al. [41] used two estimates of OHC change and the Allan et al. [24] reconstruction of ERB alongside state-of-the-art model simulations to gain insights into variations in EEI since 1960. Ocean heating rates were estimated using the Met Office Statistical Ocean Reanalysis (MOSORA) and the ORAS4 dynamical ocean reanalysis. MOSORA is novel in its use of global covariances to estimate spatially complete information from the sparse historical observations. These covariances are first estimated using a climate model and then re-estimated from the analysis once the available observations have been ingested. This process of mapping the observations and re-estimating the covariances is repeated in an iterative process designed to converge on the observed global covariances. Despite the stark methodological differences, MOSORA and ORAS4 displayed a remarkable similar time history of global OHC change. However, this may partly be a result of them using essentially the same input observations from EN3 [45].

Smith et al. [41] illustrated the utility of cross validating ERB and OHC change data sets by highlighting an inconsistency in the implied heating rates during the transition from being dominated by ship-based XBT measurements to an Argo-dominated ocean observing system. The large variability in ocean heating rates during the 2000s was not present in either the reconstruction of ERB or the model simulations of EEI during this period. In addition, there was no evidence of corresponding modulations in the rate of global sea level rise as would be expected through the influence on ocean thermal expansion. Thus, the authors concluded that the ocean heating rates were spurious and potentially the result of changes in ocean sampling and/or unresolved data biases between XBT and Argo temperature measurements. Cheng and Zhu [52] reported that the transition in ocean sampling characteristics introduced an artificial jump in OHC change around 2001–2003, in agreement with Smith et al. [41].

## Conclusions

Satellite measurements of variations in ERB and in situ measurements of OHC change represent independent and highly complementary data sets. The satellite data offer higher temporal and spatial resolution with lower sampling error estimates (0.1 Wm^−2^ on annual time series). However, the calibration accuracies of the space-borne instruments are not sufficient to provide absolute values for EEI, and while current generation platforms are relatively stable, the sensor drift could still be up to 0.5 Wm^−2^ per decade. On the other hand, OHC changes are based on direct measurement of temperature to a very high absolute accuracy (of order 0.01 K), but single-point profile observations (although they are typically representative of a much larger volume, as exploited by mapping methods) in a turbulent ocean present a challenging sampling problem and are associated with uncertainties of ±0.5 Wm^−2^ on annual timescales. The most recent Argo-based OHC change estimates suggest a decadal uncertainty of only 0.1 Wm^−2^, illustrating the utility of OHC estimates for providing a strong constraint on the absolute value of EEI on these timescales. Climate model simulations suggest that at subannual timescales, other elements of the planetary heat budget start to play a substantial role [4], and that this may place a hard limit on the utility of OHC change for monitoring shorter-term variations in EEI.

The first priority for the climate research community is to sustain the existing CERES and Argo observations to extend the record of these independent EEI estimates. Uncertainty in satellite ERB measurements may be reduced in future through better absolute calibration of sensors and refining estimates of total solar irradiance, which represent the leading order uncertainty terms. However, given the potential for OHC estimates to “anchor” EEI on longer timescales, satellite sensor stability is also an important priority. During the Argo era, reducing uncertainty in OHC estimates requires more comprehensive sampling of the oceans, including below 2000 m, the ice-covered regions and marginal seas. The development of a deep Argo array will play a key role in refining estimates of OHC change [53]. Future research is needed to determine the relative importance of these regions in the planetary energy budget, and ocean and climate model simulations are likely to play a substantial role in this. Efforts to extend both ERB and OHC estimates back into the twentieth century are also needed, although the uncertainties are likely to remain large relative to the Argo/CERES era. Systematic assessment of mapping algorithms and refinement of XBT bias corrections are a key priority for improving our understanding of the ability of OHC estimates to provide constraints on the time evolution of EEI.

## References

[1] von Schuckmann K, Palmer MD, Trenberth KE, Cazenave A, Chambers D, Champollion N, Hansen J, Josey SA, Loeb N, Mathieu P-P, Meyssignac B, Wild M. An imperative to monitor Earth’s energy imbalance. Nature Clim Change. 2016; doi:10.1038/nclimate2876.

[2] Loeb NG, Kato S, Su W, Wong T, Rose FG, Doelling DR, Norris JR, Huang X (2012). Advances in understanding top-of-atmosphere radiation variability from satellite observations. Surv Geophys.

[3] Loeb NG, Lyman JM, Johnson GC, Allan RP, Doelling DR, Wong T, Soden BJ, Stephens GL (2012). Observed changes in top-of-the-atmosphere radiation and upper-ocean heating consistent within uncertainty. Nat Geosci.

[4] Palmer MD, McNeall DJ (2014). Internal variability of Earth’s energy budget simulated by CMIP5 climate models. Env Res Lett.

[5] Trenberth KE, Fasullo JT, Balmaseda MA (2014). Earth’s energy imbalance. J Clim.

[6] Rhein M, Rintoul SR, Aoki S, Campos E, Chambers D, Feely RA, Gulev S, Johnson GC, Josey SA, Kostianoy A, Mauritzen C, Roemmich D, Talley LD and Wang F. Observations: Ocean. In: Climate Change 2013: The Physical Science Basis. Contribution of Working Group I to the Fifth Assessment Report of the Intergovernmental Panel on Climate Change [Stocker, T.F., D. Qin, G.-K. Plattner, M. Tignor, S.K. Allen, J. Boschung, A. Nauels, Y. Xia, V. Bex and P.M. Midgley (eds.)]. Cambridge University Press, Cambridge, United Kingdom and New York, NY, USA; 2013

[7] Loeb NG, Wielicki BA, Doelling DR, Smith GL, Keyes DF, Kato S, Manalo-Smith N, Wong T (2009). Toward optimal closure of the Earth’s top-of-atmosphere radiation budget. J Clim.

[8] Roemmich D, Church J, Gilson J, Monselesan D, Sutton P, Wijffels S (2015). Unabated planetary warming and its ocean structure since 2006. Nat Clim Chang.

[9] Maher N, Sen Gupta A, England MH (2014). Drivers of decadal hiatus periods in the 20th and 21st centuries. Geophys Res Lett.

[10] Risbey JS, Lewandowsky S, Langlais C, Monselesan DP, O’Kane TJ, Oreskes N (2014). Well-estimated global surface warming in climate projections selected for ENSO phase. Nature Clim. Change.

[11] Roberts CD, Palmer MD, McNeall D, Collins M (2015). Quantifying the likelihood of a continued global warming hiatus. Nature Clim. Change.

[12] Wijffels S, Roemmich D, Monselesan D, Church J, Gilson J. Ocean temperatures chronicle the ongoing warming of Earth. Nat Clim Chang. 2016;6:116–8. doi:10.1038/nclimate2924.

[13] Palmer MD, McNeall DJ, Dunstone NJ (2011). Importance of the deep ocean for estimating decadal changes in Earth’s radiation balance. Geophys Res Lett.

[14] Kandel, R. Surv Geophys (2012) 33: 337. doi:10.1007/s10712-011-9162-y

[15] Arking A, Vemury S (1984). The NIMBUS 7 ERB data set: a critical analysis. J Geophys Res.

[16] Wielicki BA, Wong T, Allan RP, Slingo A, Kiehl JT, Soden BJ, Gordon CT, Miller AJ, Yang S-K, Randall DA, Robertson F, Susskind J, Jacobowitz H. Evidence for large decadal variability in the tropical mean radiative energy budget. Science. 2002;295:841–4. 10.1126/science.1065837 11823638

[17] Kopp G, Lawrence G, Rottman G (2005). The total irradiance monitor (TIM): science results. Solar Phys.

[18] Loeb NG, Kato S, Loukachine K, Smith NM (2005). Angular distribution models for top-of-atmosphere radiative flux estimation from the clouds and the Earth’s radiant energy system instrument on the *Terra* satellite. Part I: Methodology J Atmos Oceanic Technol.

[19] Fasullo J, Trenberth K (2008). The annual cycle of the energy budget. Part II: meridional structures and poleward transports. J Clim.

[20] Stephens GL, Vane DG, Boain RJ, Mace GG, Sassen K, Wang Z, Illingworth AJ, O’Connor EJ, Rossow WB, Durden SL, Miller SD, Austin RT, Benedetti A, Mitrescu C, the CloudSat Science Team (2002). The CloudSat mission and the A-TRAIN: a new dimension to space-based observations of clouds and precipitation. Bull Am Met Soc.

[21] Winker DM, Pelon J, Coakley JA, Ackerman SA, Charlson RJ, Colarco PR, Flamant P, Fu Q, Hoff RM, Kittaka C, Kubar TL, Le Treut H, McCormick MP, Mégie G, Poole L, Powell K, Trepte C, Vaughan MA, Wielicki BA (2010). The CALIPSO mission: a global 3D view of aerosols and clouds. Bull Amer Meteor Soc.

[22] Wong, T., P. W. Stackhouse, Jr., D. P. Kratz, and A. C. Wilber, 2009: Earth radiation budget at top-of-atmosphere [in "State of the Climate in 2008"]. Peterson, T. C., and M. O. Baringer eds., Bull. Amer. Meteor. Soc., 90:8, S33-S34. doi:10.1175/BAMS-90-8-StateoftheClimate

[23] Johnson GC, Lyman JM, Loeb NG (2016). Improving estimates of Earth’s energy imbalance. Nat Clim Chang.

[24] Allan RP, Liu C, Loeb NG, Palmer MD, Roberts M, Smith D, Vidale P-L (2014). Changes in global net radiative imbalance 1985–2012. Geophys Res Lett.

[25] Abraham JP, Baringer M, Bindoff NL, Boyer T, Cheng LJ, Church JA, Conroy JL, Domingues CM, Fasullo JT, Gilson J, Goni G, Good SA, Gorman JM, Gouretski V, Ishii M, Johnson GC, Kizu S, Lyman JM, Macdonald AM, Minkowycz WJ, Moffitt SE, Palmer MD, Piola AR, Reseghetti F, Schuckmann K, Trenberth KE, Velicogna I, Willis JK. A review of global ocean temperature observations: implications for ocean heat content estimates and climate change. Rev Geophys. 2013;51:450–83. doi:10.1002/rog.20022.

[26] Desbruyères D, McDonagh EL, King BA (2016). Observational advances in estimates of oceanic heating. Current Climate Change Reports.

[27] Boyer T, Domingues CM, Good SA, Johnson GC, Lyman JM, Ishii M, Gouretski V, Willis JK, Antonov J, Wijffels S, Church JA, Cowley R, Bindoff NL (2016). Sensitivity of global upper-ocean heat content estimates to mapping methods, XBT bias corrections, and baseline climatologies. J Clim.

[28] Lyman JM, Good SA, Gouretski VV, Ishii M, Johnson GC, Palmer MD, Smith DM, Willis JK (2010). Robust warming of the global upper ocean. Nature.

[29] Palmer M, Antonov J, Barker P, Bindoff N, Boyer T, Carson M, Domingues C, Gille S, Gleckler P, Good S, Gouretski V, Guinehut S, Haines K, Harrison D, Ishii M, Johnson G, Levitus S, Lozier S, Lyman J, Meijers A, von Shuckmann K, Smith D, Wijffels S, Willis J. Future observations for monitoring global ocean heat content in proceedings of OceanObs’09: sustained ocean observations and information for society (Vol. 2), Venice, Italy, 21–25 September 2009, Hall, J, Harrison DE, Stammer D, Eds., ESA Publication WPP-306, 2010. doi:10.5270/OceanObs09.cwp.68.

[30] Carton JA, Santorelli A (2008). Global decadal upper-ocean heat content as viewed in nine analyses. J Clim.

[31] Palmer MD, Roberts CD, Balmaseda M, Chang Y-S, Chepurin G, Ferry N, Fujii Y, Good SA, Guinehut S, Haines K, Hernandez F, Köhl A, Lee T, Martin MJ, Masina S, Masuda S, Peterson KA, Storto A, Toyoda T, Valdivieso M, Vernieres G, Wang O, Xue Y. Ocean heat content variability and change in an ensemble of ocean reanalyses. Clim Dyn. 2015. doi:10.1007/s00382-015-2801-0.

[32] Xue Y, Balmaseda MA, Boyer T, Ferry N, Good S, Ishikawa I, Kumar A, Rienecker M, Rosati AJ, Yin Y. A comparative analysis of upper ocean heat content variability from an ensemble of operational ocean reanalyses. J Clim. 2012. 25:6905–29.

[33] Domingues CM, Palmer MD (2015). The IQuOD initiative: towards an international quality controlled ocean database. CLIVAR exchanges no. 67.

[34] Gouretski V, Koltermann KP (2007). How much is the ocean really warming?. Geophys Res Lett.

[35] Domingues CM, Church JA, White NJ, Gleckler PJ, Wijffels SE, Barker PM, Dunn JR (2008). Improved estimates of upper-ocean warming and multi-decadal sea-level rise. Nature.

[36] Cheng L, Zhu J, Cowley R, Boyer T, Wijffels S (2014). Time, probe type and temperature variable bias corrections to historical expendable bathythermograph observations. J Atmos Ocean Technol.

[37] Cowley RW, Wijffels S, Cheng LJ, Boyer TP, Kizu S (2013). Biases in expendable bathythermograph data: a new view based on historical side-by-side comparison. J Atmos Ocean Technol.

[38] Palmer MD, Haines K, Tett SFB, Ansell TJ (2007). Isolating the signal of ocean global warming. Geophys Res Lett.

[39] Good SA, Martin MJ, Rayner NA (2013). EN4: quality controlled ocean temperature and salinity profiles and monthly objective analyses with uncertainty estimates. Journal of Geophysical Research: Oceans.

[40] Levitus S, Antonov JI, Boyer TP, Locarnini RA, Garcia HE, Mishonov AV (2009). Global ocean heat content 1955–2008 in light of recently revealed instrumentation problems. Geophys Res Lett.

[41] Smith DM, Allan RP, Coward AC, Eade R, Hyder P, Liu C, Loeb NG, Palmer MD, Roberts CD, Scaife AA. Earth’s energy imbalance since 1960 in observations and CMIP5 models. Geophys Res Lett. 2015; doi:10.1002/2014GL062669. 10.1002/2014GL062669 PMC4459179 26074649

[42] Johnson GC, Lyman JM, Boyer T, Domingues CM, Ishii M, Killik R, Monselesan D, Wijffels SE (2016). Global oceans: ocean heat content. In state of the climate in 2015. Bull Am Meteorol Soc.

[43] Balmaseda MA, Hernandez F, Storto A, Palmer MD, Alves O, Shi L, Smith GC, Toyoda T, Valdivieso M, Barnier B, Behringer D, Boyer T, Chang Y-S, Chepurin GA, Ferry N, Forget G, Fujii Y, Good S, Guinehut S, Haines K, Ishikawa Y, Keeley S, Köhl A, Lee T, Martin MJ, Masina S, Masuda S, Meyssignac B, Mogensen K, Parent L, Peterson KA, Tang YM, Yin Y, Vernieres G, Wang X, Waters J, Wedd R, Wang O, Xue Y, Chevallier M, Lemieux J-F, Dupont F, Kuragano T, Kamachi M, Awaji T, Caltabiano A, Wilmer-Becker K, Gaillard F. The ocean reanalyses intercomparison project (ORA-IP). J Oper Oceanogr. 2015;7:81–99.

[44] Lyman JM, Johnson GC (2008). Estimating annual global upper-ocean heat content anomalies despite irregular in situ sampling. J Clim.

[45] Ingleby B, Huddleston M (2007). Quality control of ocean temperature and salinity profiles—historical and real-time data. J Mar Syst.

[46] Wong T, Wielicki BA, Lee RB, Smith GL, Bush KA, Willis JK (2006). Reexamination of the observed decadal variability of the earth radiation budget using altitude-corrected ERBE/ERBS Nonscanner WFOV data. J Clim.

[47] Willis JK, Roemmich D, Cornuelle B (2004). Interannual variability in upper ocean heat content, temperature, and thermosteric expansion on global scales. J Geophys Res.

[48] Trenberth KE, Fasullo JT (2010). Tracking Earth’s energy. Science.

[49] Church JA, White NJ, Konikow LF, Domingues CM, Cogley JG, Rignot E, Gregory JM, van den Broeke MR, Monaghan AJ, Velicogna I (2011). Revisiting the Earth’s sea-level and energy budgets from 1961 to 2008. Geophys Res Lett.

[50] Dee DP, Uppala SM, Simmons AJ, Berrisford P, Poli P, Kobayashi S, Andrae U, Balmaseda MA, Balsamo G, Bauer P, Bechtold P, Beljaars ACM, van de Berg L, Bidlot J, Bormann N, Delsol C, Dragani R, Fuentes M, Geer AJ, Haimberger L, Healy SB, Hersbach H, Hólm EV, Isaksen L, Kållberg P, Köhler M, Matricardi M, McNally AP, Monge-Sanz BM, Morcrette J-J, Park B-K, Peubey C, de Rosnay P, Tavolato C, Thépaut J-N, Vitart F (2011). The ERA-Interim reanalysis: configuration and performance of the data assimilation system. Q.J.R. Meteorol Soc.

[51] Balmaseda MA, Trenberth KE, Källén E (2013). Distinctive climate signals in reanalysis of global ocean heat content. Geophys Res Lett.

[52] Cheng L, Zhu J (2014). Artifacts in variations of ocean heat content induced by the observation system changes. Geophys Res Lett.

[53] Johnson GC, Lyman JM, Purkey SG (2015). Informing deep Argo array design using Argo and full-depth hydrographic section data. J Atmos Ocean Technol.

